# Test–retest reliability of BSP, a battery of tests for assessing spatial cognition in visually impaired children

**DOI:** 10.1371/journal.pone.0212006

**Published:** 2019-04-18

**Authors:** Sara Finocchietti, Giulia Cappagli, Giuseppina Giammari, Elena Cocchi, Monica Gori

**Affiliations:** 1 Unit for Visually Impaired People, Center for Human Technologies, Fondazione Istituto Italiano di Tecnologia, Genoa, Italy; 2 Centro regionale per l’ipovisione in età evolutiva, IRCCS Scientific Institute “E. Medea”, Bosisio Parini, Lecco, Italy; 3 Istituto David Chiossone per Ciechi ed ipovedenti ONLUS, Genova, Italy; University of Nevada Reno, UNITED STATES

## Abstract

Blind individuals are particularly dependent on their hearing for defining space. It has been found that both children and adults with visual impairments can struggle with complex spatial tasks that require a metric representation of space. Nonetheless the variability of methods employed to assess spatial abilities in absence of vision is wide, especially in the case of visually impaired children. For this reason, it would be necessary to define a battery of tests that appropriately assess different aspects of spatial perception and to investigate its reliability in order to provide a standard assessment of spatial abilities not only in experimental but also in clinical settings. The aim of this study is to determine the test–retest reliability of a battery of six spatial tasks (BSP, Blind Spatial Perception) and provide the first gold standard for assessing spatial cognition deficits in visually impaired children. Thirty visually impaired children aged 6–17 participated in two identical sessions, at a distance of 10 weeks, in which they performed six spatial tasks: auditory bisection, auditory localization, auditory distance discrimination, auditory reaching, proprioceptive reaching, and general mobility. Test–retest reliability was assessed using the test-retest scatter plots, intra-class correlation coefficient (ICC), and coefficient of variation (CV). The results showed good-to-excellent reliability for all six tests, with average ICC values ranging from 0.77 to 0.89 and average CV values ranging from 3.44% to 15.27%. In conclusion, the newly proposed battery (BSP) results as a reliable tool to identify spatial impairments in visually impaired children.

## Introduction

It is well known that visual experience affects the maturation of spatial cognition in the brain, indeed visually impaired individuals can show enhanced [1–5] as well as impaired [6–13] spatial abilities depending on the spatial aspect considered and on the task used to tag that specific spatial aspect. Although the development of spatial representation is essential in everyday life for numerous human activities [14], to date only few experimental methods have been proposed to assess how spatial representation develop in visually impaired individuals. These methods led to mixed results [15, 16], as some studies indicate that congenital blindness determines a developmental delay in sound localization abilities and motor response to sound [6, 17, 18], while other studies demonstrate that children with a visual disability correctly discriminate and identify differences in sound source location [19].

The lack of standard and validated behavioral measures to evaluate spatial abilities in blind children clearly emerge from these studies. Providing a reliability measure of spatial tests might be essential in order to adopt them in clinical settings, e.g. to assess the efficacy of rehabilitation interventions. In order to have a comprehensive view about the acquisition of spatial abilities in the blind child, it would indeed be necessary to develop a battery of behavioral tests assessing different and complementary aspects of spatial cognition in the intact domains (hearing and touch). To this end, in the present study we evaluated the reliability of a test battery comprising six spatial tests already presented in previous studies from our research group [7–10, 12] aimed at evaluating spatial cognition in a group of visually impaired children. The tests assess different spatial competencies: the ability to estimate the topographical representation of single (auditory localization task) or multiple (auditory bisection and distance tasks) sound sources and the ability to reach sonorous (auditory reaching task) or proprioceptive (proprioceptive reaching task) targets embedded in space. We also added a spatial task assessing basic functional mobility (general mobility task) by adapting for children a clinical measure typically used to assess basic functional mobility in adult patients (Timed Up and Go Test [20]). All these tests were grouped together in a test battery named BSP, Blind Spatial Perception.

The results of the reliability analysis are presented and their relevance is discussed in the context of the validation of the rehabilitation procedures used within the European project ABBI project. The project aimed at developing an innovative rehabilitation device for visually impaired children based on the implicit link between action and perception. Indeed the main outcome of the project has been the validation of a sonorous bracelet (ABBI, Audio Bracelet for Blind Interactions) with a clinical trial in which the mentioned battery of tests was used to assess post-training advancements in spatial competence in a group of visually impaired children aged 6–17 years old [21].

## Methods

### Sample size

The sample size for a test–retest reliability study depends on the target level of reliability ρ the minimally acceptable level of reliability ρ0, and the number of repetitions of the measurement π. The target level of reliability ρ is measured as intraclass correlation coefficient. The sample size is also assuming that the values for type I and type II errors (ε1 and ε2) are fixed on the typical values of 0.05 and 0.20, respectively [22]. Considering the number of measurements as 2, indicating one measurement per session and two different sessions, and with minimally accepted reliability r = 0.5 and expected reliability r = 0.75 [23], the necessary sample size for this experiment results to be 25 subjects. In order to account for unexpected high variability, data from 30 visually impaired children were collected.

### Participants

Thirty children aged 6–17 (17 females) participated in the study and performed the battery of tests in two identical sessions at a distance of 10 weeks. The main exams used for the functional assessment of visual abilities are light sources method and Early Treatment Diabetic Retinopathy Study with Lea Hyvarinen symbols chart (Lea Symbols 15-Line Translucent ETDRS-Style Distance Chart). The distance from the chart was 3 meters and assessment was performed with both eyes open using a backlit screen. The visual deficit of visually impaired participants has been interpreted according to the International Statistical Classification of Diseases and Related Health Problems (ICD) - 10th revision, according to which severe visual impairment (category 2) is related to visual acuity in the range of 0.5–1.3 LogMAR and complete blindness (category 3, 4, 5) is related to visual acuity less than 1.3 LogMAR to light perception. The children enrolled for the present study either reported a severe visual impairment (n = 20) or complete blindness (n = 10). We verified that participants exhibited no hearing impairment using the EarTest 1.0 software running on a standard pc. Clinical details of the participants are presented in Table 1. The study was approved by the local ethical committees and written informed consent from the parents was obtained in accordance with the Declaration of Helsinki.

**Table 1:** Clinical details of the participants.

#	Sex	Age	Pathology	Visual acuity
#1	M	6	Retinal distrophy	<0.01
#2	M	6	Albinism	0.05
#3	M	7	Albinism	0.10
#4	F	7	Norrie's syndrome	Light perception
#5	M	8	Retinal distrophy	0.05
#6	M	8	Achromatopsia	0.05
#7	F	9	Congenital nystagmus	0.10
#8	F	9	Optic atrophy	Light perception
#9	F	9	Cerebral astrocytoma	0.05
#10	F	9	Retinopathy prematurity	Light perception
#11	M	10	Retinal distrophy	0.07
#12	M	10	Retinoblastoma	0.01
#13	F	10	Retinopathy prematurity	NPL
#14	F	10	Retinopathy prematurity	Light perception
#15	F	11	Achromatopsia	0.10
#16	F	11	Leber’s amaurosis	0.01
#17	M	11	Lyell syndrome	0.05
#18	F	12	Retinal distrophy	0.10
#19	F	12	Optic nerves hypoplasia	0.02
#20	F	12	Congenital cataract	Light perception
#21	F	12	Leber’s amaurosis	Light perception
#22	M	13	Retinal distrophy	0.07
#23	F	13	Retinopathy Prematurity	0.10
#24	M	13	Atrophia	0.10
#25	M	15	Retinal distrophy	0.07
#26	F	15	Maculopathy	0.05
#27	F	15	Retinopathy prematurity	NPL
#28	M	16	Retinopathy prematurity	Light perception
#29	F	16	Cones dystrophy	Light perception
#30	M	17	Leber’s amaurosis	Light perception

The Table shows the gender, the age at test, the pathology and the visual acuity expressed as LogMAR.

### BSP (Blind Spatial Perception)

**Fig 1:**
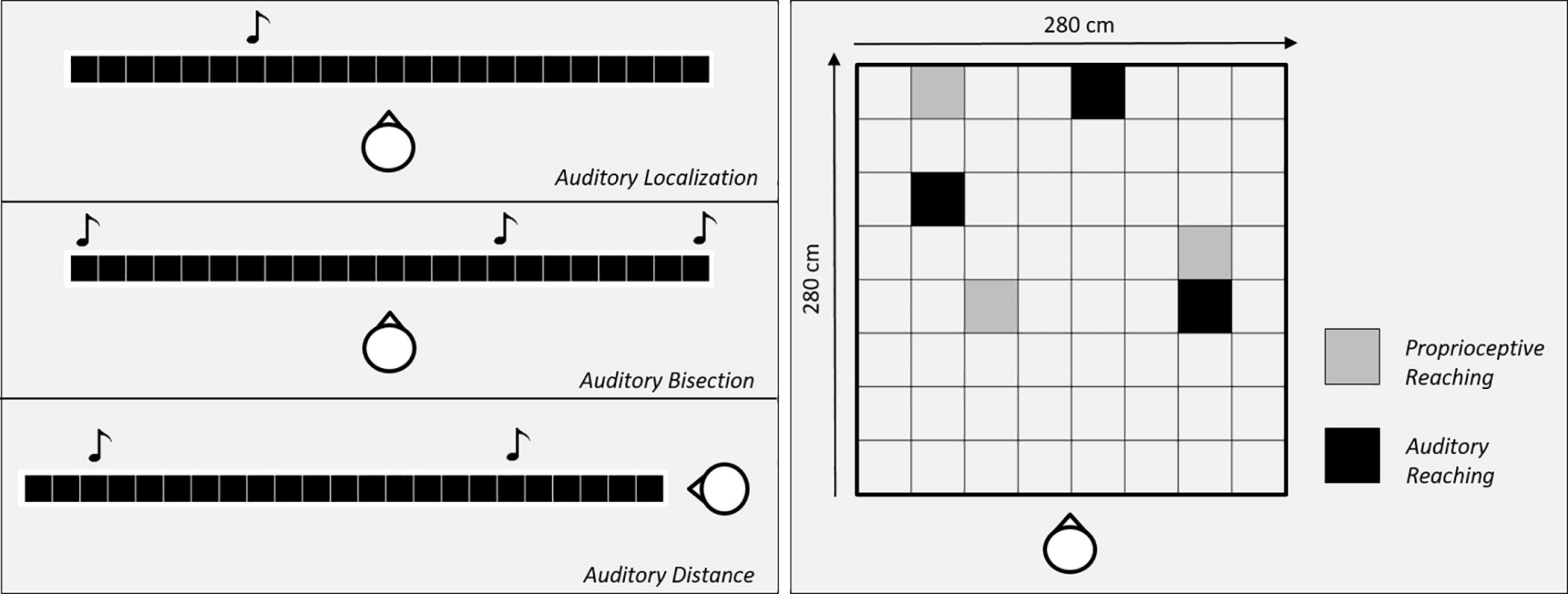
Representative description of five out of six tasks included in the BSP (Blind Spatial Perception) battery. In the left panel, the three tasks illustrated (auditory localization, auditory bisection and auditory distance) share the same experimental setup made of 23 loudspeakers embedded in a fixed array and positioned at a distance of 180 cm from the participant. In the right panel, the two tasks illustrated (auditory reaching and proprioceptive reaching) share the same experimental setup made of a carpet with a central square marked on the floor with colored tape (280 x 280 cm) on which specific positions were selected in order to perform the tasks.

The test battery BSP (Blind Spatial Perception) comprises six spatial tests that aim at evaluating auditory spatial perception across different competencies (see Fig 1). The tests have been already presented in previous studies from our research group [7–9, 21, 24, 25] and grouped together in a single battery for the purpose of the present study.

#### Auditory localization task

The test assesses children’ ability to localize a single static sound source embedded in space. According to the procedure, a single sound (500 Hz, 500 ms) is produced by one of twenty-three numbered speakers positioned frontally between -25 and + 25 degrees, in pseudo-random order. After the auditory stimulus is presented, children point to the sound location with a hand-held cane and the experimenter noted down the number associated with the indicated response. Pointing accuracy and precision are calculated offline. Localization error is defined as the mean distance in degrees between the real sound position and the indicated sound position.

#### Auditory bisection task

The test assesses children’ ability to understand the topographical representation of three stimuli embedded in space. According to the procedure, three sounds (500 Hz, 75 ms) are presented in the frontal plane successively interleaved by 500 ms intervals, the first at -25 degrees, the third at + 25 degrees, and the second at an intermediate speaker position determined by the QUEST adaptive algorithm [26] which estimates the point of subjective equality after each response, and places the next trial near that estimate. After the auditory stimuli are presented, children verbally report whether the second sound presented was closer to the first or to last sound, while the experimenter noted down the indicated response. Bisection accuracy and precision are calculated offline. Bisection error is defined as the standard deviation of the psychometric curves for each child.

#### Auditory distance task

The test assesses children’ ability to understand the topographical representation of two stimuli embedded in space. According to the procedure, two consecutive sounds (500 Hz, 500 ms) are produced by two out of the twenty-three loudspeaker array positioned between -25 and + 25 degrees in the depth dimension. After the auditory stimuli are presented, children verbally report which of the two stimuli (first or second) presented was closer in space to their own body and the experimenter noted down the indicated response. Distance accuracy and precision are calculated offline. The proportion of trials where the probe was judged closer than the standard was computed for each probe position, and data were fitted with a Gaussian error function.

#### Auditory reaching task

The test assesses children’ ability to localize and reach a single static sound source embedded in space. According to the procedure, a single sound (500 Hz) is played for five seconds in one out of three specific positions in the frontal space. After the auditory stimulus is presented, the child is asked to reach the sound source position by walking. Three different positions are randomly selected three times each, for a total of nine trials. Reaching accuracy and precision are calculated offline. Localization error is defined as the mean distance (in cm) between the real sound position and the indicated sound position.

#### Proprioceptive reaching task

The test assesses children’ ability to passively explore a motion trajectory in space and successively repeat it without external guidance. According to the procedure, the experimenter guides the child from a fixed starting position in the room toward one out of three selected positions and then back to the starting position. After the demonstration, the child is asked to reproduce the whole trajectory by his own. Three different positions are randomly selected three times each, for a total of nine trials. Reaching accuracy and precision are calculated offline. Localization error is defined as the mean distance (in cm) between the real position and the reached position.

#### General mobility task

The test assesses children’ speed of walk as a general measure of mobility [21]. This test was adopted and modified from the Timed up and go task [20]. The child is given a signal “ready, 1, 2, 3, and go.” On the go cue, the child is asked to walk for three meters marked by a tactile stimulus on the floor, turn around of 180° with the help of the experimenter, and walk back to the starting position. The time in seconds is recorded from the “go” cue to when the child reaches the starting position.

## Data analysis and statistics

All values are presented as mean and standard error of the mean (SEM). P values smaller than 0.05 were regarded as significant. The data were compared using repeated measures analysis of variance (RM ANOVA) with session (one, two) as factors. RM-ANOVAs were performed using Statistica, version 5.1 (StatSoft Inc., USA). ICCs and Bland Altman analysis were performed using custom-made excel spreadsheets (available upon request). Test–retest reliability intended as the ability of a measure applied twice upon the same respondents to produce the same ranking on both occasions (also referred to as stability over time), has been assessed using multiple methods described below, along with guidelines to help interpreting the results:

*• Intraclass correlation coefficient (ICC)*: it measures the relative homogeneity within sessions in relation to the total observed variation between sessions and it is measured on a scale of 0 to 1, where 1 represents perfect reliability with no measurement error while 0 indicates no reliability. For this analysis, a two-way mixed model using absolute agreement was selected, and ICC for single measurements was reported. ICC values above 0.75 are indicative of good reliability [27].
• *Coefficient of variation (CV)*: it is a measure of relative variability. Specifically it represents the standard error of measurement expressed as a percentage of the subject’s average threshold. The CV can be interpreted as the percentage of deviation from the average threshold below which 68% of the differences between sessions may be expected to lie [28].
• *Bland–Altman agreement analysis*: it is a graphical method to represent the average difference between two sessions or methods of measurement (the 'bias'), from which the limits of agreement (LA) can be derived as the average difference +/-1.96 times the standard deviation of the differences. The LA delimits the range within which 95% of the differences between thresholds in two single sessions may be expected to lie [29]. The 95% limits of agreement is generally plotted for visual judgement of how well two sessions or methods of measurement agree, therefore the smaller the range between the two limits the better the agreement is.

## Results

### Difference between thresholds and sessions

**Fig 2:**
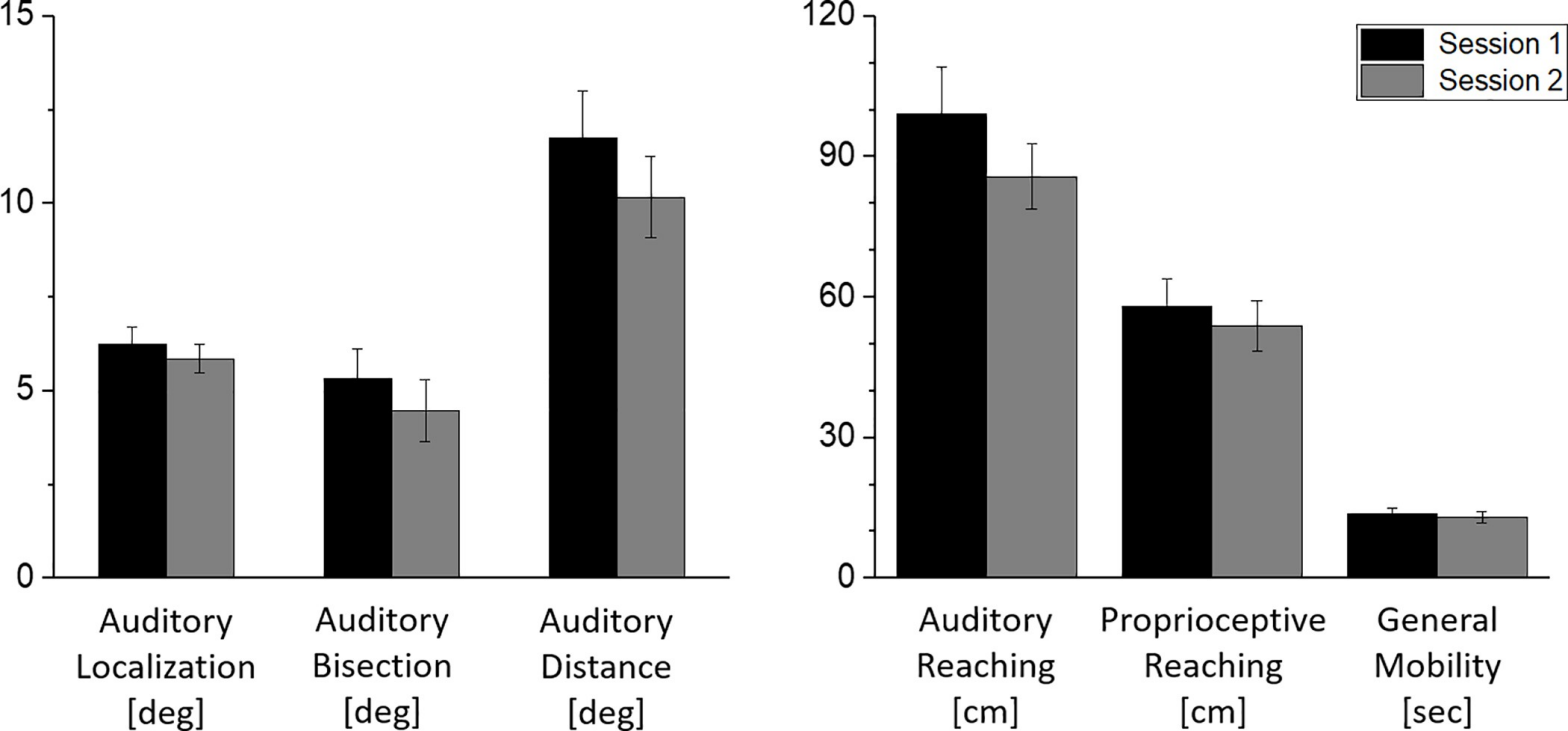
Means and differences for the six tasks included in the BSP battery. Mean (+/- standard error of the mean [SEM], N = 30) of the BSP battery assessed in two different sessions at a distance of ten weeks.

Overview means and differences in sessions for the six tests (Fig 2). The Kolmogorov–Smirnov test was used for normality assessment, and all the data were normally distributed. The ANOVA showed no statistical difference between the two sessions in all the tests performed (p > 0.1).

### Reliability of the BSP battery

**Fig 3:**
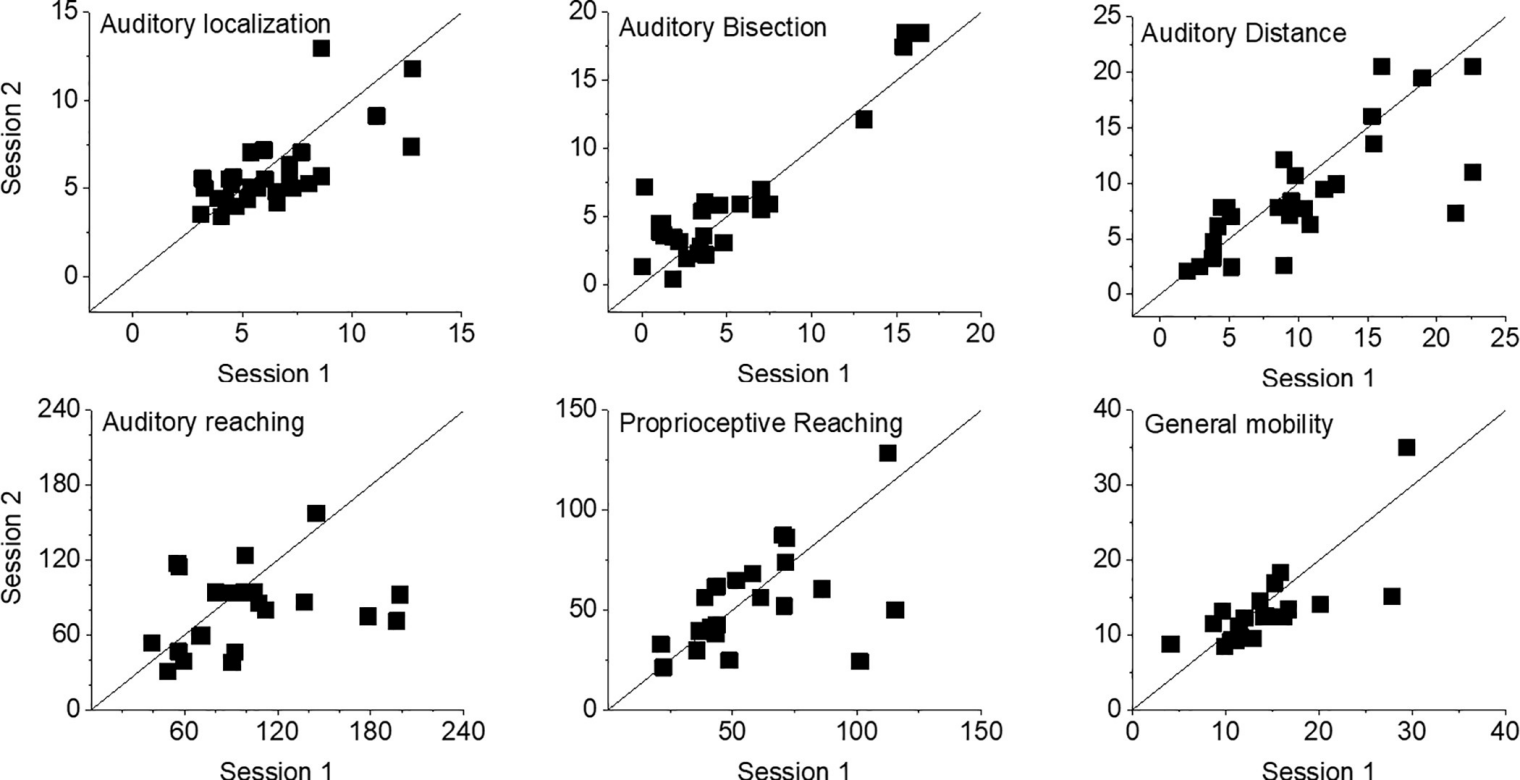
Test–retest Scatter plots for the auditory localization, auditory bisection, auditory distance, auditory reaching, proprioceptive reaching and general mobility task. Panels show scores at session 1 and session 2 for all the individual test paradigms in 30 subjects. Test–retest points would coincide perfectly if they do not deviate from the 45° line.

Fig 3 shows scatter plots of test–retest values of the six tests giving impression about relative reliability. Detailed ICC and CV values are shown in Table 2. ICC values were above 0.75 and were significantly different from 0.5, thus indicating an overall good to excellent reliability. The highest ICC value was 0.94 for the auditory bisection task, depicting a “perfect agreement”, while the lowest was 0.75 for the auditory reaching.

**Table 2:** Detailed analysis of ICC and CV.

	ICC	Confidence Interval (ICC)	Coefficient variation CV
Auditory localization	0.82	[0.68; 0.90]	4.69%
Auditory bisection	0.94	[0.89; 0.97]	8.83%
Auditory distance	0.90	[0.83; 0.95]	10.23%
Auditory reaching	0.75	[0.55; 0.87]	15.27%
Proprioceptive reaching	0.77	[0.60; 0.88]	5.38%
General Mobility Task	0.89	[0.78; 0.84]	3.44%

**Fig 4:**
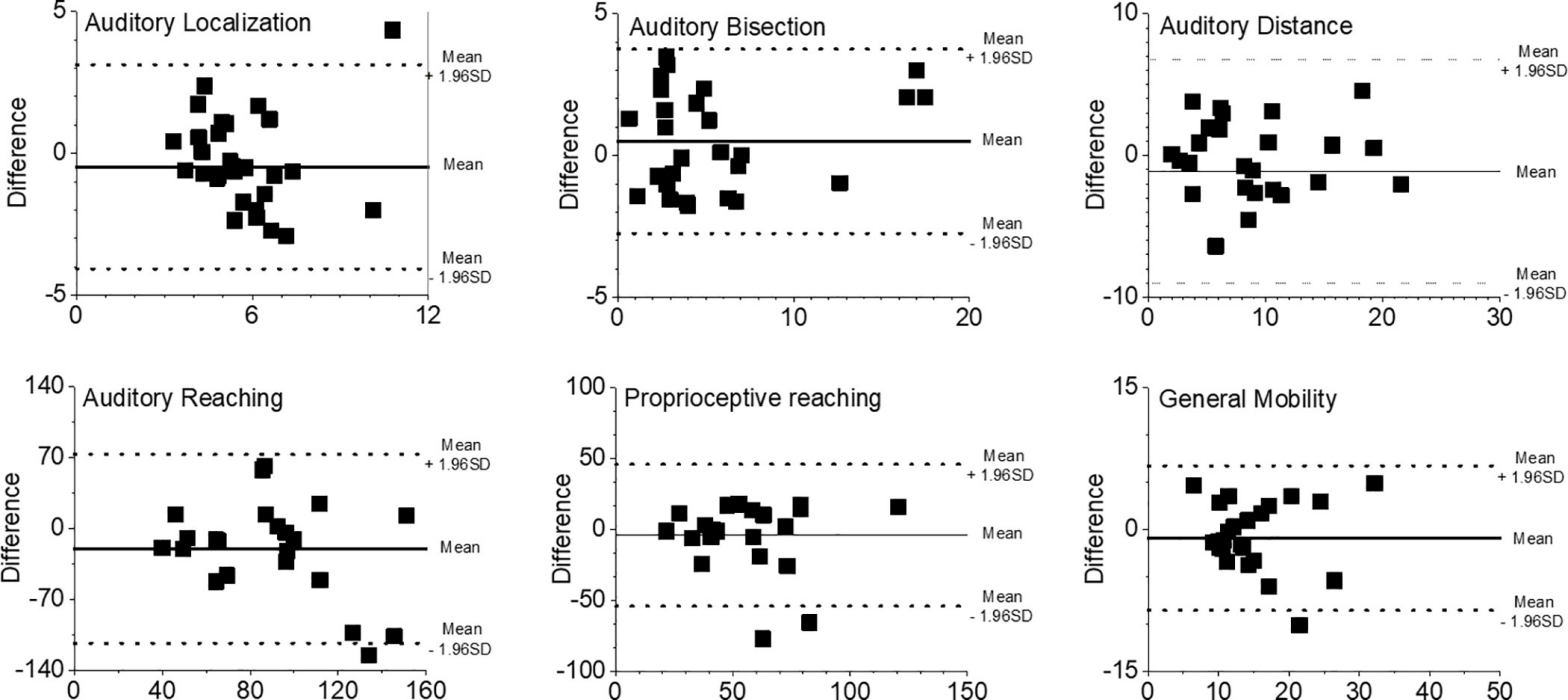
Bland–Altman plots for the auditory localization, auditory bisection, auditory distance, auditory reaching, proprioceptive reaching and general mobility task. The *solid* line indicates the mean difference between the two sessions, while the *dotted* lines indicate the limits of agreement.

Bland–Altman plots obtained during the two test sessions for each task are presented in Fig 4 and provide graphical impression of absolute reliability measures. The auditory localization task revealed an averaged (left and right) 95% confidence interval LA = -4.08 to 3.12 deg, and an absolute averaged BIAS of 0.47 deg. The auditory bisection task revealed an averaged (left and right) 95% confidence interval LA = -2.75 to 3.75 deg, and an absolute averaged BIAS of 0.49 deg. The Auditory Distance task revealed an averaged (left and right) 95% confidence interval LA = -9.04 to 6.73 deg, and an absolute averaged BIAS of 1.15 deg. The auditory reaching task revealed an averaged (left and right) 95% confidence interval LA = -113.33 to 73.56 cm, and an absolute averaged BIAS of -19.88 cm. The proprioceptive reaching task revealed an averaged (left and right) 95% confidence interval LA = -54.04 to 47.11 cm, and an absolute averaged BIAS of 3.95 cm. Finally, the general mobility task revealed an averaged (left and right) 95% confidence interval LA = -8.54 to 6.76 s, and an absolute averaged BIAS of 0.89 s. Regarding the coefficient of variation CV, presented in Table 2, values were ranging from 3.44% for the general mobility task to 15.27% for the proprioceptive reaching task.

## Discussion

As previously suggested, the acquisition of spatial abilities is of fundamental importance for children because it influences the capability to independently navigate in the environment and the disposition to engage in positive social interaction with peers [30]. Since vision provides the most accurate and reliable information about the spatial properties of the external world, the absence of visual information significantly affects spatial cognition [31, 32]. Indeed it has been suggested that blindness challenges the acquisition of self-knowledge in the environment, mainly because it prevents the development of body awareness in relation to objects and the physical world and impairs exploratory and locomotor abilities [33].

It is worth noting to point out that there aren’t gold standard methods to assess the development of spatial cognition in visually impaired individuals. This can led to confounding results about the characterization of spatial impairments in blind individuals which in turn might cause misinterpretation about the effectiveness of early rehabilitation procedures.

To fill this gap, the aim of this study was to determine the reliability of a new BSP test battery (Blind Spatial Perception) aiming at evaluating spatial perception across different but complementary competencies in visually impaired children. Reliability can be seen as the degree to which a test measures the same way each time it is used under the same condition with the same subjects Reliability can be categorized as relative or absolute [28, 34, 35]. Relative reliability refers to the degree to which individuals’ measurements or scores maintain their position relative to others. Absolute reliability refers to the degree to which individuals’ measurements or scores vary, assessed across repeated measures. Common methods used to quantify absolute reliability are the coefficient of variation, and the 95% LA proposed by Bland and Altman [28, 29, 34, 36]. All the six tests included in the BSP test battery presented good to excellent test–retest reliability, with a minimum ICC value of 0.77 for the proprioceptive reaching task and a maximum of 0.94 for the auditory bisection task. It should be noted that the differences in reliability among the different tests were, in general, modest, giving an additional strength to the battery as a comprehensive tool for assessing spatial perception in visually impaired children.

The present results indicate that the six tasks evaluated (auditory localization, auditory bisection, auditory distance, auditory reaching, proprioceptive reaching, general mobility) properly tag fundamental and complementary aspects of spatial perception, suggesting that the BSP battery could be considered a gold standard for the assessment of spatial perception in visually impaired individuals.

## Supporting information

S1 File Raw data presented by tasks. The file contains the raw data for each task performed (auditory localization, auditory bisection, auditory distance, auditory reaching, proprioceptive reaching, general mobility) on sessions one and two for each participant involved in the study.
(XLSX)
